# Laparoscopic Approach Towards Non-Communicating Functional Rudimentary Uterine Horn: A Report of Two Cases

**DOI:** 10.7759/cureus.11419

**Published:** 2020-11-10

**Authors:** Jasmina Begum, Nibedita Maharana, Shashi Shankar Behera, Manwar Ali, Sudipta Mohakud

**Affiliations:** 1 Obstetrics and Gynecology, All India Institute of Medical Sciences, Bhubaneswar, IND; 2 Obstetrics and Gynecology, The Advanced Medical Research Institute, Bhubaneswar, IND; 3 Surgery, All India Institute of Medical Sciences, Bhubaneswar, IND; 4 Radiology, All India Institute of Medical Sciences, Bhubaneswar, IND

**Keywords:** unicornuate uterus, dysmenorrhoea, rudimentary horn, laparoscopy

## Abstract

A unicornuate uterus is a relatively rare Müllerian anomaly with an incidence of 2.5-13%. It may lead to various gynecological or obstetric complications, and diagnosis can often be confusing and delayed. It is associated with varying clinical presentations depending on the presence of functional endometrium, which requires immediate surgical resection on the diagnosis.

We report two cases of the unicornuate uterus in young women who presented with severe progressive dysmenorrhoea. These cases highlight the challenges in diagnosing the condition by ultrasound, which was confirmed later by MRI. Both cases were managed by laparoscopic resection of the functional non-communicating uterine horn. On follow-up, both patients were found asymptomatic with normal menstrual cycles.

In patients of young age who present with abdominal pain, adnexal masses of unknown origin, and severely painful periods, we should consider Müllerian duct anomalies as one of the differential diagnoses. Early and proper preoperative diagnosis of these cases is essential to prevent complications and to offer adequate treatment. Operative laparoscopy is an excellent alternative to laparotomy for their management, particularly in young unmarried girls.

## Introduction

A unicornuate uterus is a relatively rare malformation caused by abnormal or failed development of one of the paired Müllerian ducts, and its incidence has been reported to be 2.5%-13% [1,2]. Müllerian duct anomalies occur because of complete or incomplete agenesis, defective fusion, or resorption failure. The unicornuate uterus results from aplasia or atresia of one side of the Müllerian tube. Incomplete atresia of a Mullerian duct results in a rudimentary horn, which is broadly connected through a streak of tissue with the unicornuate uterus, and in 74% of the cases, there is no communication between the two endometrial cavities [3,4]. Patients are mostly asymptomatic in rudimentary horn with a non-functional and non-communicating endometrial cavity. However, a non-communicating horn with a functional endometrial cavity can cause painful menstruation, lump abdomen, and dyspareunia due to hematometra, hematosalpinx, or endometriosis following retrograde menstruation. It may be associated with malformations of the upper urinary tract as well [5]. These uterine anomalies are either diagnosed incidentally or when they present with gynecologic and obstetric complications [6]. Imaging studies like ultrasound and MRI are found to be useful in the diagnosis as well as for surgical planning and anticipation of the amount of myometrial connection [7]. Resection of the rudimentary horn is the definitive treatment modality. We describe two cases of non-communicating functional rudimentary horns that were successfully managed laparoscopically.

## Case presentation

Case 1

A 16-year-old unmarried girl presented to the outpatient Department of Obstetrics and Gynaecology of our institute with complaints of severe dysmenorrhoea and vomiting. She had attained menarche at the age of 13 years, and since then, she had experienced progressively increasing dysmenorrhoea. Her menstrual cycles were regular at an interval of 28-30 days, and with a monthly flow of three to five days. On general examination, her vitals were normal. Her secondary sexual characters were well developed. On abdominal examination, there was tenderness in the right iliac fossa. On pelvic examination, the vulva was normal with an intact hymen. Per rectal examination, the uterus was normal in size with an approximately 4 x 4 cm palpable mass in the right adnexal region. The patient was having horizontal nystagmus, microcornea, and iris coloboma. Transabdominal ultrasound showed features of bicornuate uterus with the right-side paraumbilical ectopic kidney. MRI of the abdomen and pelvis was subsequently done, which revealed a unicornuate uterus with a right-side non-communicating horn and hematometra along with a right-side paraumbilical ectopic kidney. Electroencephalogram (EEG) and MRI brain and spine were found to be normal.

**Figure 1:**
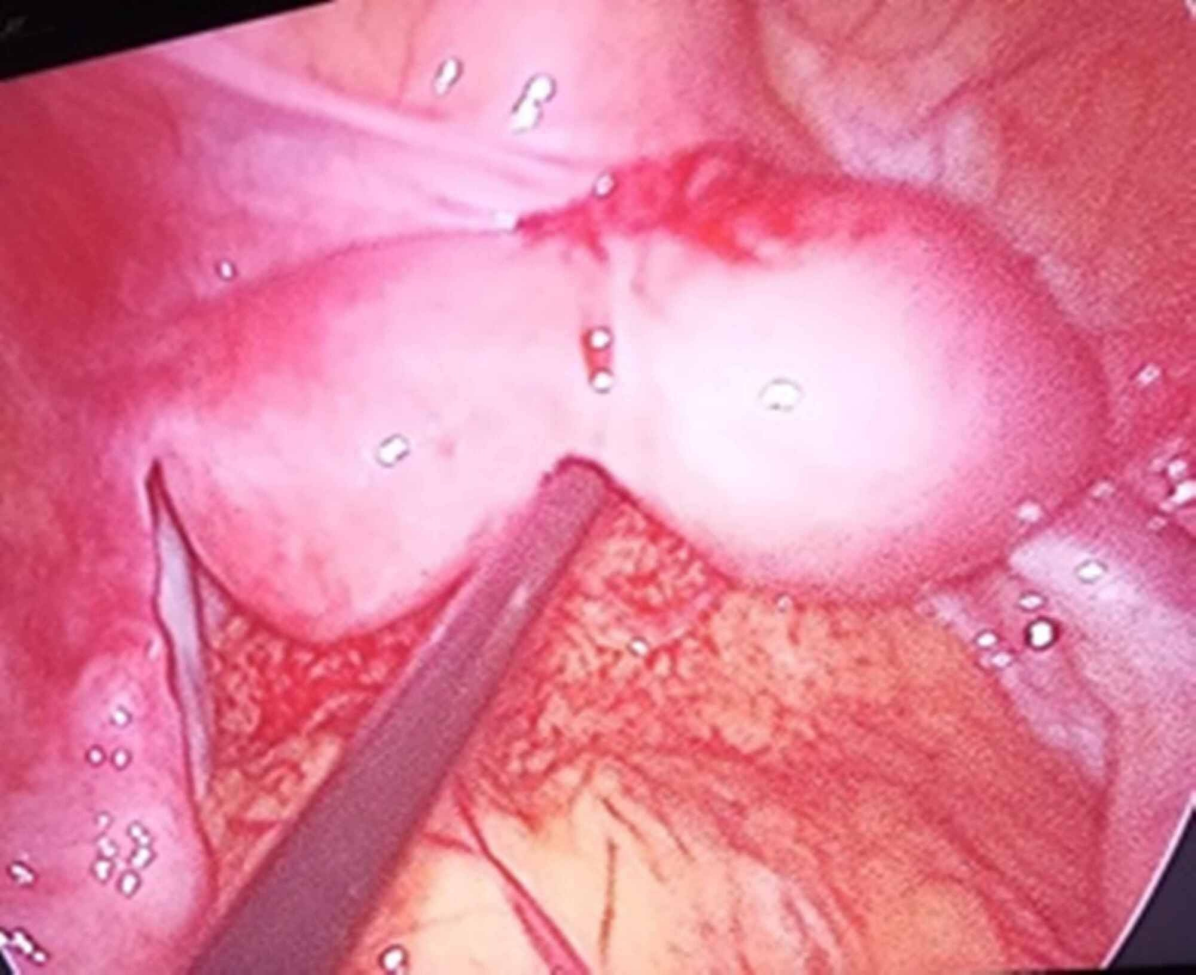
Case 1 intraoperative laparoscopic image 1 Laparoscopic imaging of the right-side rudimentary horn of the unicornuate uterus

**Figure 2:**
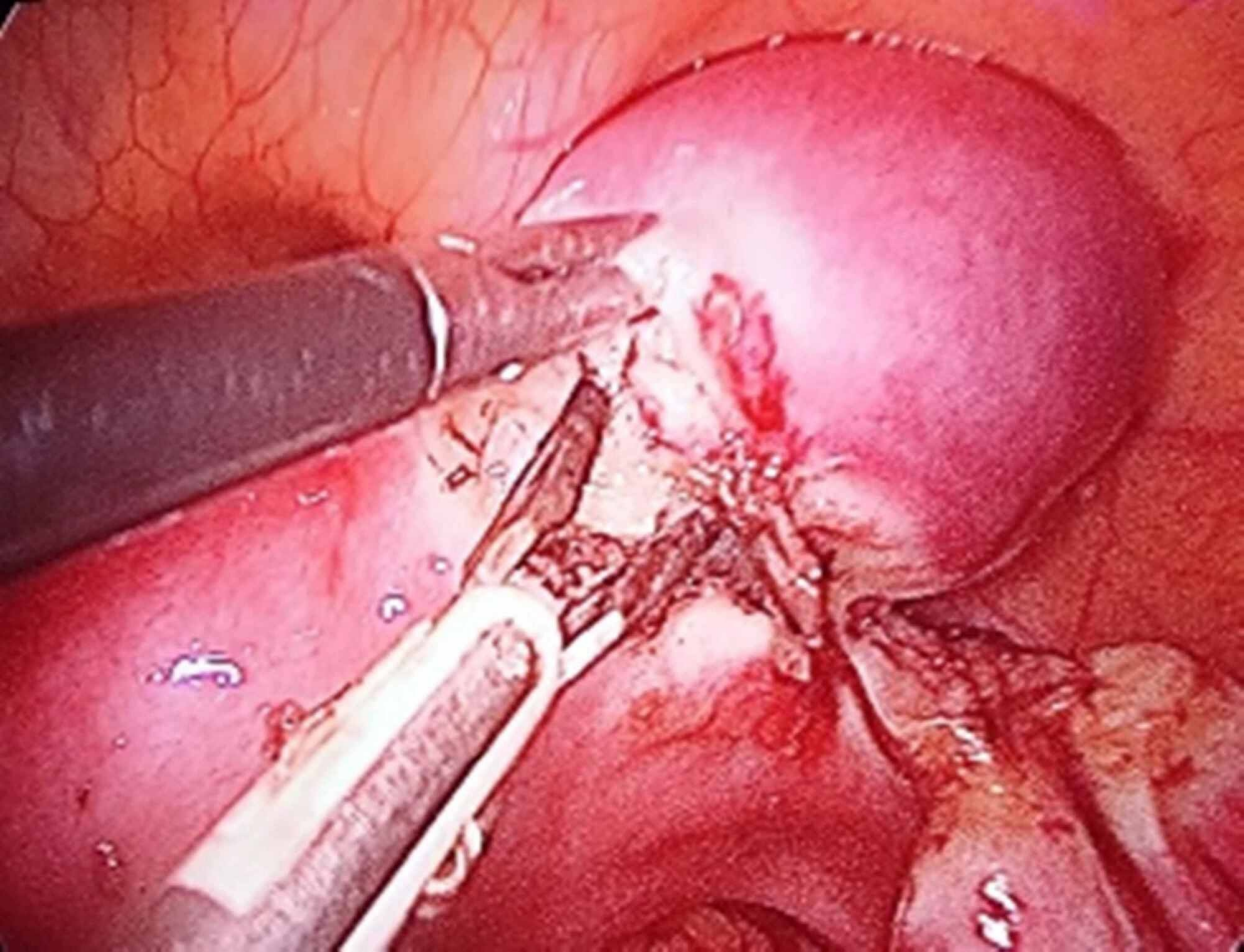
Case 1 intraoperative laparoscopic image 2 Laparoscopic resection of the right-side rudimentary horn of the unicornuate uterus

The patient was planned for laparoscopy as consent for hysteroscopy could not be obtained. Intraoperative findings during laparoscopy were as follows: a unicornuate uterus with a distended right rudimentary horn. The left round ligament was attached to the left uterine cornual region; however, the right round ligament arose from the rudimentary horn. The non-communicating rudimentary horn was attached by a thick band of tissue to the uterus. The left-side fallopian tube was healthy. The right-sided fallopian tube was short, thickened, and beaded. Bilateral ovaries were normal. No evidence of endometriotic lesion was seen in the pelvis. Laparoscopically, the rudimentary horn was resected, right salpingectomy was performed (Figures 1, 2), and this was followed by the repair of the resected myometrium by interrupted square sutures. During resection, adequate care was taken to avoid entry into the endometrium of the uterus. For proper identification of ectopic kidney and prevention of inadvertent ureteric injury, we also had a surgeon as standby. Histopathological study of the rudimentary horn revealed functional endometrium. The postoperative period was uneventful, and the patient was discharged on the third postoperative day. The patient came for a follow-up after her next menses and was found to be asymptomatic.

Case 2

A 21-year-old unmarried female was admitted to the emergency ward with complaints of severe dysmenorrhoea. She had attained menarche at 14 years. Her previous menstrual cycles had been regular, with an average flow. She also had progressively increasing dysmenorrhoea. On admission, her vitals were normal and there was diffuse tenderness on abdominal examination. On rectal examination, the uterus could not be delineated and there was tenderness on the right side. Ultrasonography revealed the presence of two endometrial cavities with collection inside the right-side cavity suggesting hematometra. MRI confirmed the presence of non-communicating right horn with hematometra.

**Figure 3:**
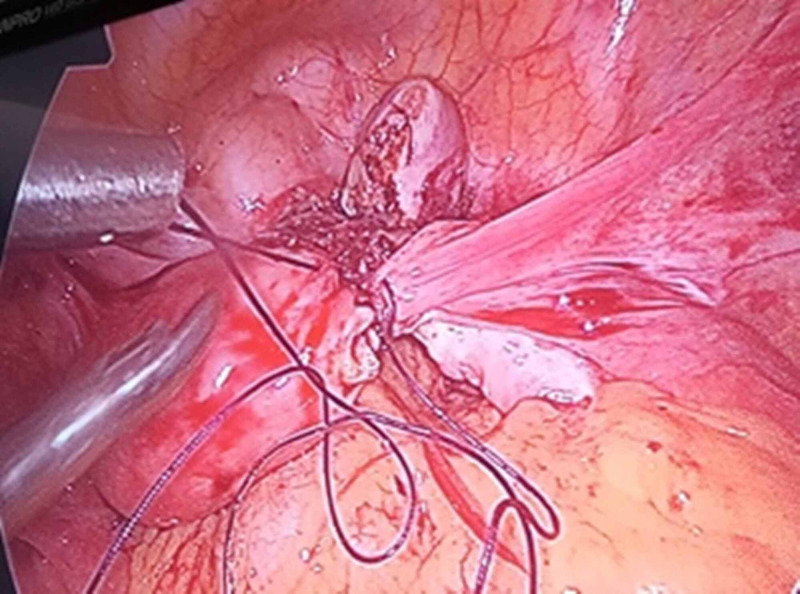
Case 2 intraoperative laparoscopic image Laparoscopic resection of the right-side rudimentary horn and repairing of resected myometrium

**Figure 4:**
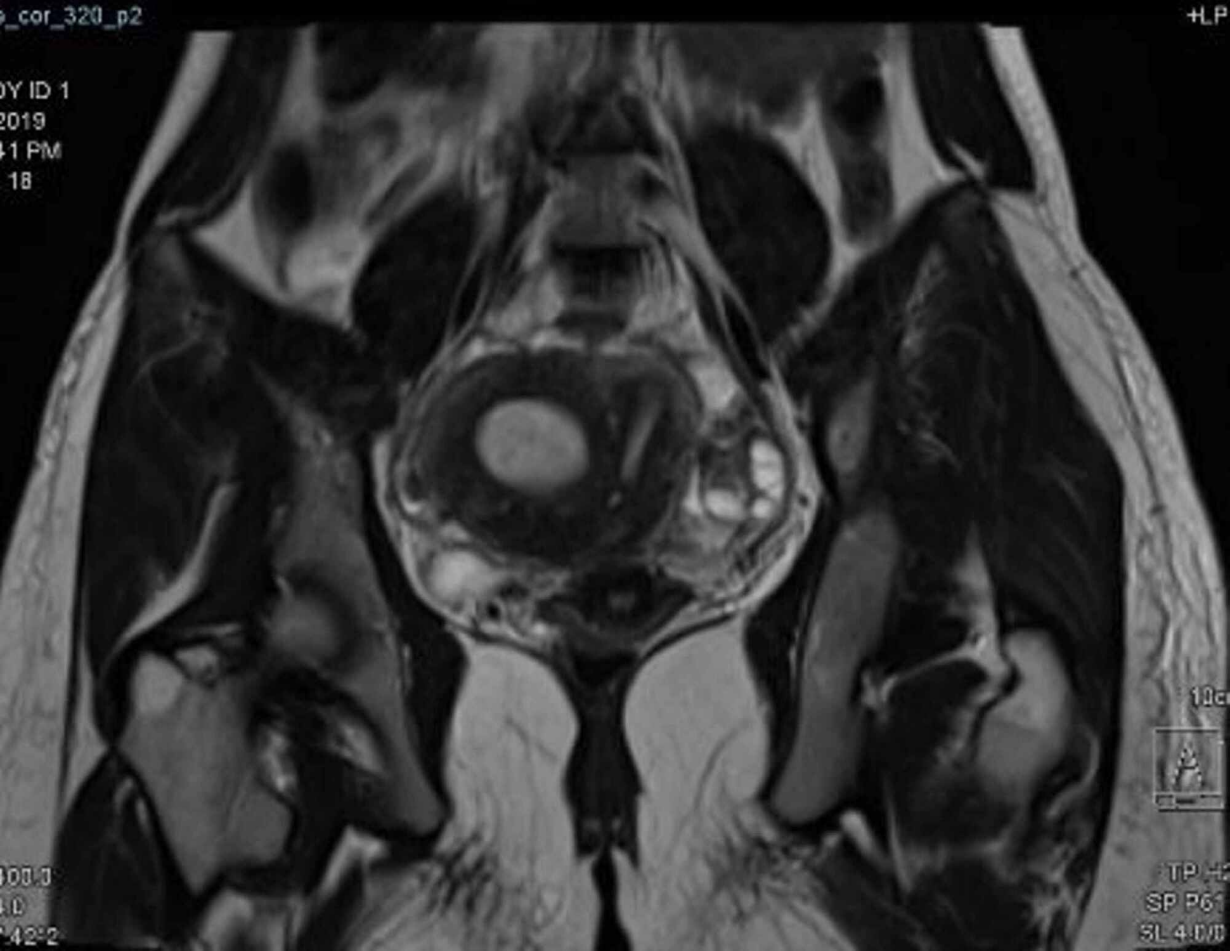
MRI of the pelvis Pelvic MRI: T2 signal MRI indicated the presence of the right-side functional rudimentary horn with hematometra in a unicornuate uterus. Both ovaries were demonstrated MRI: magnetic resonance imaging

The patient was planned for diagnostic and therapeutic hystero-laparoscopy after obtaining informed consent. On hysteroscopic examination, the uterine cavity was tubular, and the left-sided tubal ostium was visualized with the absence of right-side ostium. Laparoscopy confirmed the presence of a rudimentary horn on the right side, which was attached to the uterus by a broad band of tissue. We proceeded with the resection of the right rudimentary horn with right salpingectomy without disturbing the integrity of the endometrial cavity of the left side followed by the repair of the myometrium by interrupted square sutures. The surgeon’s help was also sought intraoperatively for proper identification of the ureters. The patient was discharged on the third postoperative day (Figures 3, 4). At the subsequent follow-up, the patient was found to be asymptomatic with regular menstrual cycles.

## Discussion

The actual incidence of the unicornuate uterus is difficult to ascertain as many patients remain asymptomatic due to the non-functional noncommunicating horn [1,2]. The recent European Society of Human Reproduction and Embryology (ESHRE)/European Society for Gynaecological Endoscopy (ESGE) classification system for Müllerian anomalies classifies Class U0 as normal uteri, Class U1 as dysmorphic uteri, Class U2 as septate, Class U3 as bicorporeal, Class U4 as hemi-uterus, Class U5 as atrophic, and Class U6 as unclassified [8]. According to this, a hemi-uterus with a rudimentary functional horn is Class U4 (a), whereas Class U4 (b) is a hemi-uterus with a rudimentary non-functional horn. The presence of functional endometrium in the non-communicating horn is the key factor for complications such as acute abdomen and hematometra as seen in our case. It can also lead to complications like ectopic pregnancy, torsion, and endometriosis if left untreated [9].

The diagnosis of the unicornuate uterus is difficult with conventional ultrasonography as demonstrated in both of our cases. However, MRI is the accepted gold standard for the detection of Müllerian anomalies [10-12]. Intravenous pyelogram (IVP) can also be a diagnostic tool to rule out any associated renal anomaly, but its role is limited in the presence of MRI [13]. Preoperative MRI and IVP are essential to plan out the surgical proceedings as they can give a better overview of the pelvic anatomy and the extent of the myometrial connection. Hysteroscopy should also be performed for diagnostic purposes and to exclude vaginal or cervical anomalies. It is also helpful in the visualization of the plane of dissection between the rudimentary horn and uterus by trans-illumination [14]. Hysteroscopy was done in our second case, which confirmed the non-communicating horn and helped us in the resection of the horn. Once diagnosed, surgical excision of the non-communicating functional rudimentary horn is recommended to avoid potential complications like hematometra, endometriosis, and future pregnancy-related complications [15]. Laparoscopic approaches have comparable outcomes to laparotomy; however, with emerging skills in minimally invasive surgery and the added advantages of shorter hospital stay, small incisions, and less postoperative pain, laparoscopy has become the first choice for experienced hands despite the challenging and complex nature of the procedure [16-18]. In our cases, we had performed a careful preoperative evaluation of both patients to detect anatomical subtype, attachment type, and associated malformations of the upper urinary tract. We succeeded in the laparoscopic removal of the rudimentary horn along with the same-side fallopian tube without any complications. This was possible because we took utmost care in defining the anatomical landmarks, proper cleavage planes, followed careful hemostasis, identified the ipsilateral ureter, and traced its pelvic course. We also performed simultaneous hysteroscopy to evaluate the uterine cavity to find the relationship with the rudimentary horn. In both cases, we sutured the myometrial defect to avoid any potential uterine rupture in future pregnancies since both patients were young girls. The postoperative period was uneventful, and on subsequent follow-ups, both patients were found to be asymptomatic with normal menstrual cycles.

## Conclusions

Unicornuate uterus, though uncommonly seen, should be considered with a high index of suspicion in cases of acute abdomen and progressive dysmenorrhoea in the post-menarcheal group. Early diagnosis of this condition is essential to prevent gynecological or obstetric complications. Proper preoperative evaluation for potential urinary tract anomalies is the most important step to avoid intraoperative complications. High levels of laparoscopic skills are required for the effective management of the unicornuate uterus with a non-communicating functional horn.

## References

[1] Grimbizis GF, Camus M, Tarlatzis BC, Bontis JN, Devroey P (2001). Clinical implications of uterine malformations and hysteroscopic treatment results. Hum Reprod Update.

[2] Acién P (1997). Incidence of Müllerian defects in fertile and infertile women. Hum Reprod.

[3] Falcone T, Gidwani G, Paraiso M, Beverly C, Goldberg J (1997). Anatomical variation in the rudimentary horns of a unicornuate uterus: implications for laparoscopic surgery. Hum Reprod.

[4] Behrens M, Licata M, Lee JY (2020). The infected hematometra in a rudimentary noncommunicating horn misdiagnosed as pelvic mass: a case report. Int J Surg Case Rep.

[5] Jayasinghe Y, Rane A, Stalewski H, Grover S (2005). The presentation and early diagnosis of the rudimentary uterine horn. Obstet Gynecol.

[6] Rani A, Kumari M, Shipra Shipra (2015). A case of non-communicating uterine horn containing functional endometrium. Gynecol Obstet (Sunnyvale).

[7] Spitzer RF, Kives S, Allen LM (2009). Case series of laparoscopically resected noncommunicating functional uterine horns. J Pediatr Adolesc Gynecol.

[8] Grimbizis GF, Gordts S, Di Spiezio Sardo A (2013). The ESHRE/ESGE consensus on the classification of female genital tract congenital anomalies. Hum Reprod.

[9] Matalliotakis IM, Goumenou AG, Koumantakis GE, Neonaki MA, Koumantakis EE, Arici A (2002). Pulmonary endometriosis in a patient with unicornuate uterus and noncommunicating rudimentary horn. Fertil Steril.

[10] Wu MH, Hsu CC, Huang KE (1997). Detection of congenital müllerian duct anomalies using three-dimensional ultrasound. J Clin Ultrasound.

[11] Pellerito JS, McCarthy SM, Doyle MB, Glickman MG, DeCherney AH (1992). Diagnosis of uterine anomalies: relative accuracy of MR imaging, endovaginal sonography, and hysterosalpingography. Radiology.

[12] Vallerie AM, Breech LL (2010). Update in Müllerian anomalies: diagnosis, management, and outcomes. Curr Opin Obstet Gynecol.

[13] Obeidat RA, Aleshawi AJ, Tashtush NA, Alsarawi H (2019). Unicornuate uterus with a rudimentary non-communicating cavitary horn in association with VACTERL association: case report. BMC Women’s Health.

[14] Kriplani A, Agarwal N (2001). Hysteroscopic and laparoscopic guided miniaccess hemihysterectomy for non-communicating uterine horn. Arch Gynecol Obstet.

[15] Karabulut A, Herek D, Demirlenk S, Calıskan S, Sevket O (2012). An unusual form of unicornuate uterus with noncommunicating rudimentary horn: case report and review of the literature. J Gynecol Surg.

[16] Medeiros LR, Rosa DD, Silva FR, Silva BR, Rosa MI (2011). Laparoscopic approach of a unicornuate uterus with noncommunicating rudimentary horns. ISRN Obstet Gynecol.

[17] Tekani H, Karthik G (2016). Unicornuate uterus with a functional non-communicating horn in a parous woman. J Obstet Gynaecol India.

[18] Mabrouk M, Arena A, Zanello M, Raimondo D, Seracchioli R (2020). Unicornuate uterus with noncommunicating functional horn: diagnostic workup and laparoscopic horn amputation. Fertil Steril.

